# In Situ Crosslinking Bionanocomposite Hydrogels with Potential for Wound Healing Applications

**DOI:** 10.3390/jfb10040050

**Published:** 2019-11-14

**Authors:** Federica Leone, Melike Firlak, Kirsty Challen, Wayne Bonnefin, Barbara Onida, Karen L. Wright, John G. Hardy

**Affiliations:** 1 Department of Chemistry, Lancaster University, Lancaster LA1 4YB, UK; federica.leone@polito.it (F.L.); m.firlak@gmail.com (M.F.); 2 Department of Biomedical and Life Sciences, Lancaster University, Lancaster LA1 4YG, UK; 3 Politecnico di Torino, Department of Applied Science and Technology, Corso Duca Degli Abruzzi 24, 10129 Turin, Italy; 4 Lancashire Teaching Hospitals NHS Trust, Emergency Department, Royal Preston Hospital, Sharoe Green Lane, Preston PR2 9HT, UK; kirsty.challen@lthtr.nhs.uk; 5 Advanced Medical Solutions Group PLC., Premier Park, 33 Road One, Winsford Industrial Estate, Winsford, Cheshire CW7 3RT, UK; wayne.bonnefin@admedsol.com; 6 Materials Science Institute, Lancaster University, Lancaster LA1 4YB, UK

**Keywords:** in situ forming, injectable, hydrogel, polysaccharide, bionanocomposite, wound healing

## Abstract

In situ forming hydrogels are a class of biomaterials that can fulfil a variety of important biomedically relevant functions and hold promise for the emerging field of patient-specific treatments (e.g., cell therapy, drug delivery). Here we report the results of our investigations on the generation of in situ forming hydrogels with potential for wound healing applications (e.g., complex blast injuries). The combination of polysaccharides that were oxidized to display aldehydes, amine displaying chitosan and nanostructured ZnO yields in situ forming bionanocomposite hydrogels. The physicochemical properties of the components, their cytotoxicity towards HaCat cells and the in vitro release of zinc ions on synthetic skin were studied. The in situ gel formation process was complete within minutes, the components were non-toxic towards HaCat cells at functional levels, Zn^2+^ was released from the gels, and such materials may facilitate wound healing.

## 1. Introduction

Biomaterials to facilitate wound healing are designed to cover wounds, prevent infection and help injured tissues to repair and regenerate with an improved rate of healing [1]. A variety of different materials have been investigated for such applications, which are generally hydrophilic, porous and swellable, in various morphologies (including fibers, films, foams and hydrogels) [1]. The materials may be degradable (useful for internal wounds) and/or loaded with therapeutically active molecules/particles (e.g., drugs and antimicrobials) [1]. Hydrogels are a particularly appealing class of materials because they are hydrophilic polymer networks that are swollen with water, and when applied to a wound site they are effective in absorbing exudates and protecting wounds from secondary infection [2,3], which may be of particular benefit in challenging situations (e.g., battlefields) [4]. Several methods have been investigated for the preparation of in situ forming hydrogels (e.g., chemical/photochemical crosslinking) [5,6,7,8,9,10,11] and in situ forming biodegradable hydrogels without potentially toxic catalysts are appealing for their potential to cover/fill patient-specific wounds with varying dimensions (e.g., unusually shaped and/or large wounds not suitable for the more traditional dressings) [4]. In the long term it may be possible to 3D print patient specific materials for wound coverage [12], although this is most likely applicable under sterile conditions in a hospital instead of battlefields.

A multitude of different chemistries have been investigated for the preparation of in situ forming hydrogels with their own advantages/disadvantages [13]. ‘Click’ chemistry has had a significant impact on the preparation of natural/synthetic polymer-based biomaterials [14,15,16]. Seminal early work from Hawker and co-workers described the use of copper-catalyzed click reactions to form hydrogels for biomedical applications; however, this requires washing with copper chelating agents after the gel is formed to remove the toxic copper [17]. The subsequent development of copper-free click reactions (e.g., strain-promoted azide-alkyne cycloadditions, thiol-ene chemistry, thiol-yne chemistry) has enabled the generation of hydrogels for biomedical applications and is discussed in depth in a number of papers and reviews [18,19,20,21]; it is noteworthy that ‘Click’ chemistry has been applied to prepare polysaccharide-based hydrogels [22].

It is possible to generate constitutionally dynamic mixtures of products via Schiff base formation between either aldehydes or ketones and amines displayed on peptides or proteins in the biological milieu [23,24], and such inherently adaptable chemistry can generate in situ crosslinking hydrogels that are clinically relevant soft biomaterials for personalized medicine (e.g., for wound healing). Injectable hydrogels based on polysaccharides and/or PEG derivatives displaying aldehydes and hydrazides have been reported by various groups [25,26,27].

Polysaccharides are a common component of hydrogels used for biomedical applications because they are cheap, degradable, their properties can be tuned (e.g., as a function of molecular weight), and they tend to be non-immunogenic [9,10,11]. Pectin (a component of cell walls of plants) [9] and hyaluronic acid (HA, a component of the extracellular matrix) [26,27,28,29,30,31] are highly hydrophilic because of the carboxylic acids displayed on their backbones, and their backbones can be oxidized to generate reactive aldehyde moieties that can react with amines forming Schiff base linkages that can form sample spanning hydrogels in periods from seconds to minutes dependent on the concentration of reactive amines/aldehydes in three-dimensional space [32,33,34,35].

Chitosan (CS, a partially deacetylated derivative of chitin) is an abundant waste product of the food industry that is biocompatible, degradable and displays antimicrobial activity because of the amines displayed on its backbone [10,11]. Chitosan is an appealing alternative to other antimicrobial polymers used in wound treatments (e.g., polyhexamethylene biguanide, PHMB) [36,37,38,39] because the amines also underpin the Schiff base crosslinks that enable gel formation. Moreover, depending on how rapidly the in situ forming wound covering is applied, the chitosan may play a role in staunching blood flow due to its hemostatic nature [40].

ZnO is listed by the Food and Drug Administration (FDA) as a Generally Recognized as Safe (GRAS) substance, due to its low toxicity, biocompatibility, biodegradability and antimicrobial properties. ZnO particles with various properties can be prepared by a variety of methods and are appealing for a variety of biomedical applications (e.g., drug delivery, tissue engineering and regenerative medicine) [41,42,43,44,45]. A recent study concluded that the use of nanostructured ZnOs would be as safe or safer than bulk ZnO due to the lower cytotoxicity of the nanostructured ZnO towards keratinocytes, which is promising for wound healing applications [46].

**Figure 1:**
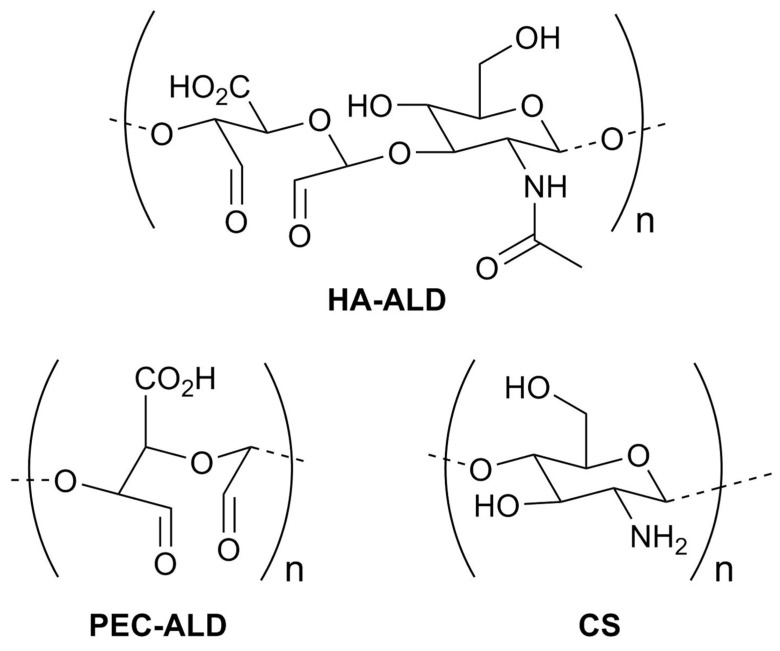
Structures of the polysaccharide derivatives studied.

Bionanocomposites are an emerging class of advanced materials composed of combinations of biopolymers with inorganic/organic nanoparticles. Here we report the generation of in situ forming hydrogels formed by imine formation between amines displayed on CS and aldehydes displayed on oxidized derivatives of HA or pectin (Figure 1). The inclusion of nanostructured ZnO inside the hydrogels was investigated to enhance the antimicrobial potential of these hydrogels for wound healing applications (in part due to the release of Zn^2+^ ions from the surface of the particles) [47]. The physicochemical properties of the bionanocomposites, the cytotoxicity of the different components towards HaCat cells, and the in vitro release of zinc ion on synthetic skin were studied and reported herein.

## 2. Results and Discussion

The in situ crosslinking hydrogels reported here are crosslinked via imine (Schiff base) formation between polysaccharides displaying aldehydes on their backbones and polysaccharides displaying amines on their backbones [25,26,27]. As noted above, the formation of such bonds is swift, potentially enabling hydrogel formation in seconds to minutes, dependent on the concentration of reactive groups in three-dimensional space [25,26,27]. Such materials have a range of potential biomedical applications, particularly when composed of polysaccharides such as chitosan, hyaluronic acid or pectin [48,49,50,51].

**Figure A1:**
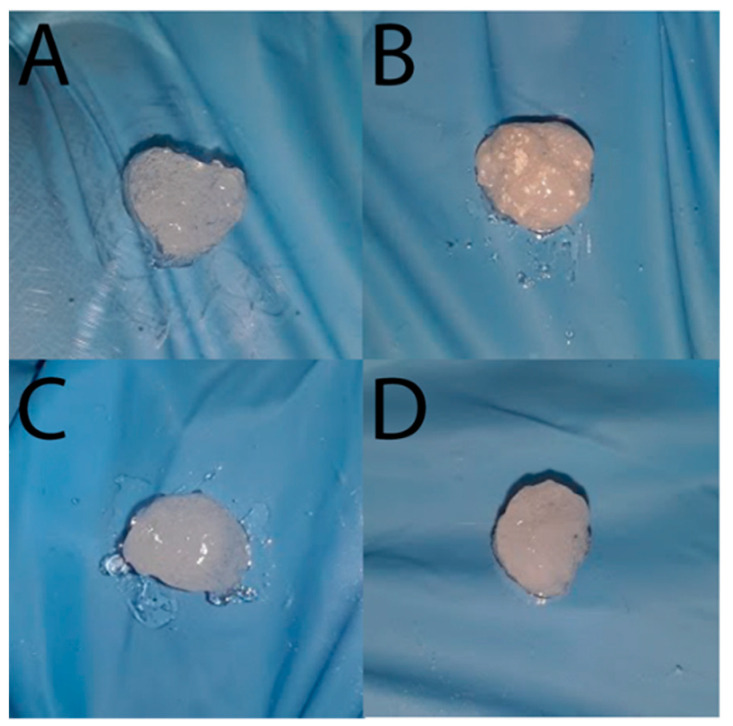
Photographs of the hydrogels studied supported on a gloved hand. (**A**) Translucent HA-ALD/CS polysaccharide gels. (**B**) Opaque HA-ALD/CS/NsZnO bionanocomposite gels. (**C**) Translucent PEC-ALD/CS polysaccharide gels. (**D**) Opaque PEC-ALD/CS/NsZnO bionanocomposite gels.

HA and pectin were oxidized with sodium periodate yielding HA-ALD and PEC-ALD, respectively, which were readily soluble at 1 wt% in phosphate buffered saline (PBS, pH 7.4); CS was readily soluble at 2 wt% in acidified water (pH 5.5). Homogeneous hydrogels were prepared by mixing the stock solutions at a volume ratio of 1:2 for HA-ALD:CS, or 1:1 for PEC-ALD:CS, with crosslinking occurring within seconds of mixing the components yielding translucent gels (due to light scattering from the CS) [52,53] and the inclusion of NsZnO (1 wt%) rendered the gels opaque (Figure A1).

**Figure 2:**
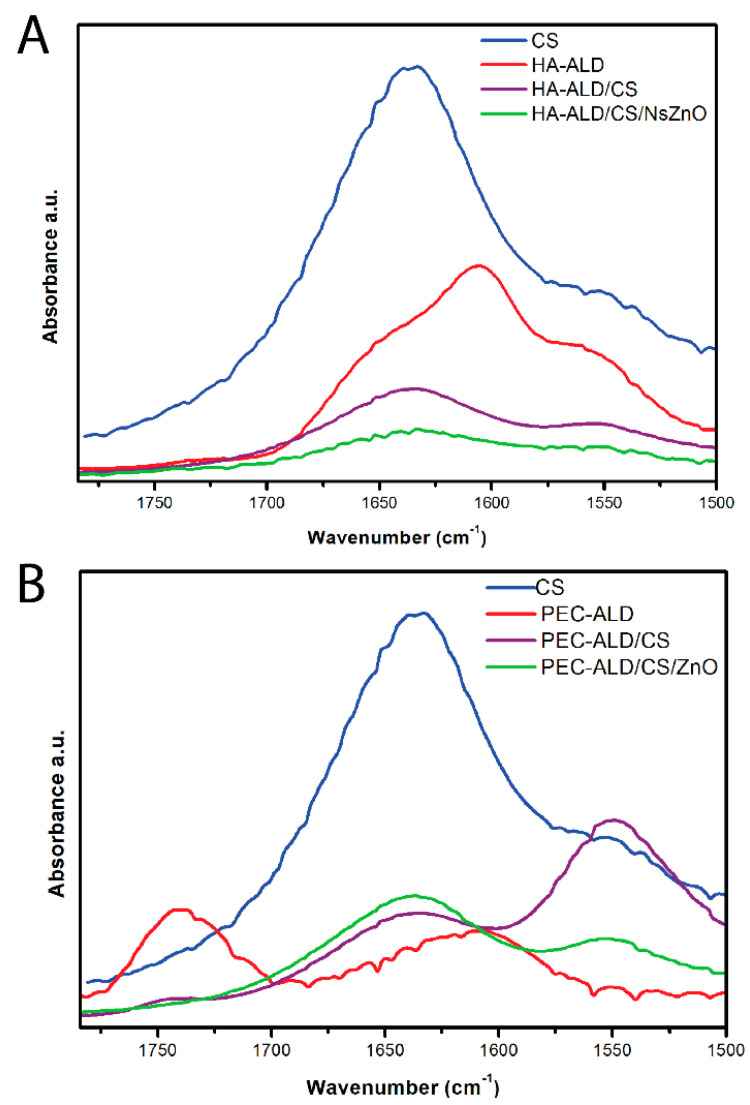
FTIR spectra. (**A**) Chitosan and HA-ALD as single components, in comparison to the polysaccharide gels and the bionanocomposite gels. (**B**) Chitosan and PEC-ALD as single components, in comparison to the polysaccharide gels and the bionanocomposite gels.

The Fourier-transform infrared (FTIR) spectra of the oxidized derivatives of hyaluronic acid (HA-ALD) and pectin (PEC-ALD) showed weak shoulders for the aldehydes at ~1720 cm^−1^. The FTIR spectra of the hydrogels without/with the NsZnO particles (Figure 2) confirm the successful crosslinking of the amines displayed on CS with HA-ALD (Figure 2A) or PEC-ALD (Figure 2B) due to the appearance of a peak at ~1550 cm^−1^ corresponding to imine bonds (this peak is somewhat masked by the amide II peaks for the acetylated amines present on HA and the 75–85% deacetylated CS). Such chemistry is readily adaptable to a variety of other polysaccharides [54], enabling its use as a platform for the generation of in situ forming and self-healing gel-based materials [55].

**Figure 3:**
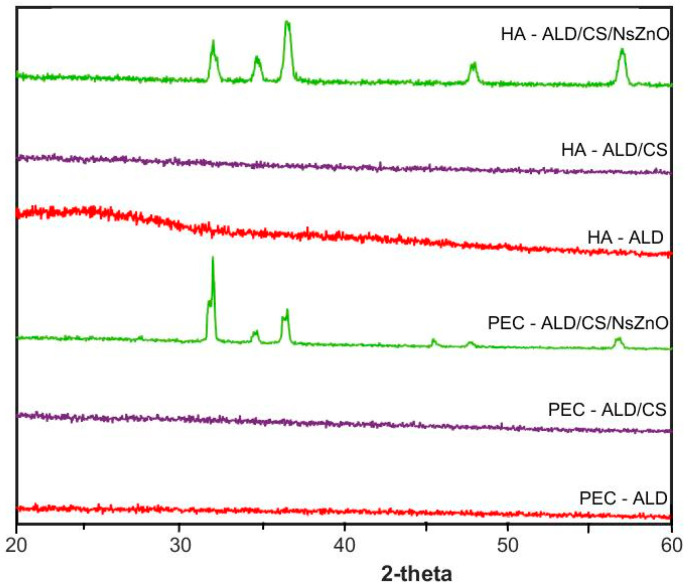
XRD patterns.

**Figure A2:**
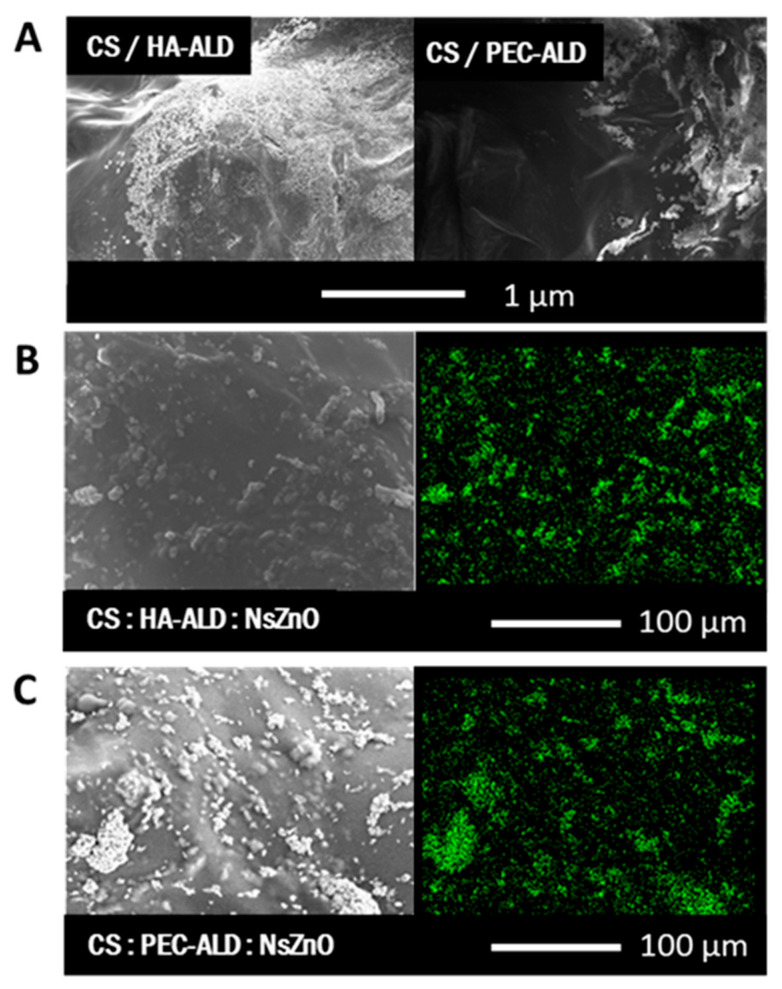
(**A**) FESEM images of the dried hydrogels (left: CS/HA-ALD polysaccharide gel; right: CS/PEC-ALD polysaccharide gel). (**B**) HA-ALD/CS/NsZnO bionanocomposite hydrogel (left: FESEM image; right: EDS elemental mapping image). (**C**) PEC-ALD/CS/NsZnO bionanocomposite hydrogel (left: FESEM image; right: EDS elemental mapping image).

The X-ray diffraction (XRD) patterns (Figure 3) of HA-ALD and PEC-ALD are amorphous, and the gels formed from these components in the absence of NsZnO are correspondingly amorphous [56]. The XRD patterns of the bionanocomposite hydrogels containing NsZnO have peaks corresponding to NsZnO that are in agreement with our previously reported data [57], with clear evidence of a crystalline hexagonal phase with a wurtzite structure: the five main reflection peaks (100), (002), (101), (102) and (110) are consistent with those of the standard card for the hexagonal phase ZnO (JCPDS ICDD 36e1451). The inclusion of the NsZnO nanoparticles in the gels is clearly evident from the presence of the wurtzitic NsZnO XRD patterns in the bionanocomposite hydrogels. The preservation of the wurtzitic ZnO crystalline structure after the dispersion of the nanoparticles in the gel matrix is significant because of the link between structure and function [41]. Importantly, scanning electron microscopy (SEM) and energy-dispersive X-ray spectroscopy (EDS) confirmed the homogeneous dispersion of the NsZnO particles inside the polymeric matrix (Figure A2). This is of interest for the antibacterial activity of these biomaterials, as the reproducibility of the diffusion of Zn^2+^ will be dependent upon the homogeneity of dispersion within the gel matrix.

**Figure A3:**
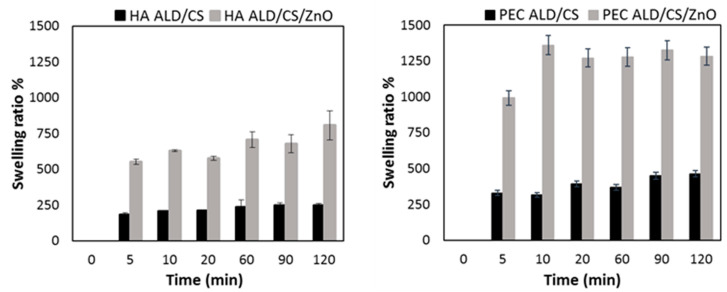
Swelling behavior of the hydrogels. Left: swelling profiles of HA-ALD/CS polysaccharide gels (black bars) and HA-ALD/CS/NsZnO bionanocomposite gels (grey bars). Right: swelling profiles of PEC-ALD/CS polysaccharide gels (black bars) and PEC-ALD/CS/NsZnO bionanocomposite gels (grey bars).

The swelling behavior of the gels without/with NsZnO in PBS was studied (Figure A3). As noted above, Schiff base formation occurs rapidly, potentially enabling hydrogel formation in seconds to minutes depending on the density of the reactive species in three-dimensional space. The swell ratio of a gel is related to the degree of crosslinking of the polymeric species; it was observed that the swell ratios of the polysaccharide components of the gels studied were stable after ~20 min. The addition of the NsZnO to the hydrogels (i.e., generation of bionanocomposite hydrogels) resulted in higher swell ratios than the hydrogels composed of polysaccharides only. The increased swell ratio is likely to be because the presence of NsZnO diminishes the number of Schiff base crosslinks that form, and the NsZnO repels the polymer chains due to electrostatic interactions, thereby increasing the pore sizes within the hydrogel. There is a significant body of literature on hydrogels crosslinked by dynamic Schiff base formation, and the swell ratios of the hydrogels reported in this manuscript fall within the broad range reported in the literature [25,26,27]. For wound healing applications, absorption of exudate is important as it diminishes risks associated with secondary infections [58]; however, we note that the swell ratio desired will be dependent on the specific paradigm in which the hydrogel will be applied (i.e., in the absence/presence of pressure applied to the wound, etc.).

**Figure 4:**
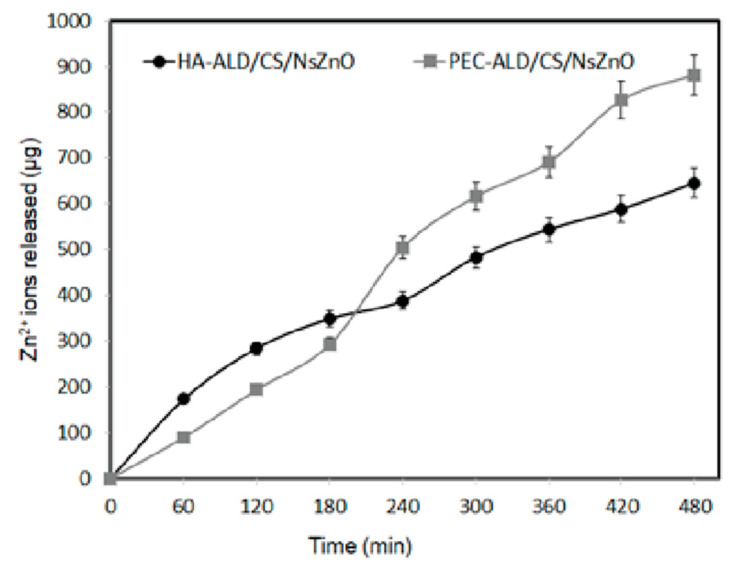
Zinc release from the bionanocomposite hydrogels. Black circles represent HA-ALD/CS/NsZnO. Grey squares represent PEC-ALD/CS/NsZnO.

The pH of wound exudate normally ranges between pH 4.8 and pH 9.8, consequently the PBS was chosen as a simple biologically relevant mimic of the non-extreme wound environment [59]. The release profiles of Zn^2+^ from the bionanocomposite hydrogels was studied over the period of 8 h (Figure 4), with release of Zn^2+^ at a rate of ~100 µg per hour for both formulations (there was no statistically significant difference between the formulations), which is potentially useful for wound healing. The importance of Zn^2+^ in our physiology (e.g., for cell membrane repair, cell proliferation, growth and immune system function) and in modulating the wound healing process is discussed in depth in an excellent review [43]. We envisage future iterations of these gels (with polysaccharides displaying varying densities of aldehyde/amine functionalities and with various sizes/morphologies of NsZnO) will enable more precise control of the amounts of Zn^2+^ delivered, which may facilitate control of the chronobiology of the wound healing process [43].

**Figure 5:**
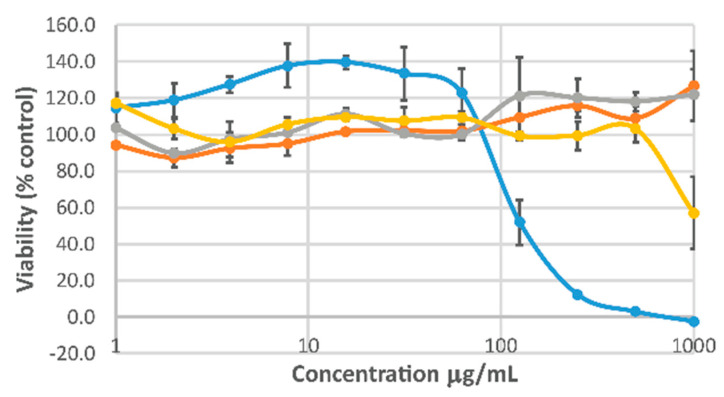
HaCaT cell viability after incubation with the components of the gel formulations assessed using a PrestoBlue^TM^ Cell Viability Assay. Chitosan M.M.W. (yellow), HA-ALD-24 (grey), PEC-ALD-24 (orange), NsZnO (blue). Data depicted are the average percentages of control (untreated cells in medium alone) ± SD, n = 3.

The immortalized human keratinocyte (HaCaT) cell line is a model cell line used to evaluate epidermal homeostasis and its pathophysiology [60] and is therefore of potential interest for wound healing paradigms. The cytotoxicity of the individual components of the gels was assessed using HaCaT cells (Figure 5) and cell viability was maintained across a broad range of concentrations for HA-ALD and PEC-ALD (in line with the literature on other cells [25,26,35,53,56]), with somewhat higher level of cytotoxicity for CS (likely to be because the cationic CS may disrupt cell membranes). The viability of HaCaT cells in the presence of ZnO has already been studied [46,61,62] and the HaCaT cells responded in a similarly dose-dependent fashion to NsZnO, consequently we believe these nanocomposite gels to have some potential for wound healing applications. We envisage future iterations of these gels (with polysaccharides displaying varying densities of aldehyde/amine functionalities and tunable amounts of NsZnO) will deliver a therapeutically relevant quantity of Zn^2+^ and thereby control the chronobiology of the wound healing process [43], which will be undertaken in collaboration with clinicians and industrial partners to guide the successful development of the materials towards clinically relevant outcomes.

## 3. Materials and Methods 

### 3.1. Materials

Unless otherwise noted, all reagents were purchased from Sigma-Aldrich, Gillingham, UK, and used as received without further purification. For cell culture, all reagents were purchased from Thermo Fisher Scientific, Morecambe, UK. HaCaT cells (immortalized human keratinocytes) were a gift from Sarah Allinson (Lancaster University), originally purchased from Lonza, Manchester, UK.

### 3.2. Synthesis of Hyaluronic Acid Displaying Aldehydes

Oxidized HA (HA-ALD) was prepared in accordance with the literature [16]. Then, 1 g of HA (MW ~2 MDa) was dissolved in ultrapure (Millipore) water (100 mL) to produce a solution with a concentration of 10 mg/mL by stirring for ~24 h. Sodium periodate (0.535 g, 2.5 mM) was added and the reaction was stirred for 24 h at room temperature in the dark. After this time, the unreacted periodate was eliminated by the addition of ethylene glycol (140 μL, 2.5 mM) and the reaction was stirred for another 1 h at room temperature in the dark. Dialysis against ultrapure water was performed using dialysis tubing (cellulose membrane, MWCO 3500) to remove the low molecular weight contaminants (exchanging the water every 2 h during the daytime for 4 days). The product (HA-ALD) was isolated by freeze-drying.

### 3.3. Synthesis of Pectin Displaying Aldehydes

Oxidized pectin (PEC-ALD) was prepared by adaptation of the literature [32]. Then, 1 g of PEC (poly-D-galacturonic acid methyl ester, MW 21–70 kDa) was dissolved in ultrapure (Millipore UK, Watford, UK) water (100 mL) to produce a solution with a concentration of 10 mg/mL by stirring for ~24 h. Sodium periodate (0.535 g, 2.5 mM) was added and the reaction was stirred for 24 h at room temperature in the dark. After this time, the unreacted periodate was eliminated by the addition of ethylene glycol (140 μL, 2.5 mM) and the reaction was stirred for another 1 h at room temperature in the dark. Dialysis against ultrapure water was performed using dialysis tubing (cellulose membrane, MWCO 3500) to remove the low molecular weight contaminants (exchanging the water every 2 h during the daytime for 4 days). The (PEC-ALD) was isolated by freeze-drying. 

### 3.4. Synthesis of NsZnO

Nanostructured (Ns) ZnO was prepared (using a soft template, Pluronic F127) and characterized in accordance with the literature [57]. An aqueous solution of Pluronic F127 (10% v/v) was prepared, and zinc acetate was then added to a concentration of 0.8 M. The resulting opalescent suspension was stirred at room temperature for 2 h, after which it was isolated by vacuum filtration and then calcinated at 500 °C for 165 min at a heating rate of 3 °C/min to remove the soft template and obtain porous zinc oxide.

### 3.5. Preparation of Chitosan Stock Solution

CS with a medium molecular weight (MW 190–310 kDa, 75–85% deacetylated) was used. A CS stock solution of 2 wt% was prepared adding the polysaccharide powder into an aqueous solution of acetic acid (99:1 volume ratio H_2_O: acetic acid). After complete dissolution, the pH was adjusted to 5.5 by drop-wise addition of 1 M sodium hydroxide, and this formulation was stirred for 24 h to ensure homogeneity.

### 3.6. Polysaccharide Hydrogel Preparation

The 1 wt% solutions of HA-ALD or PEC-ALD in PBS (pH 7.4) and a 2 wt% solution of CS in acidified water (pH 5.5) were prepared. Homogeneous hydrogels were prepared by mixing the stock solutions at a volume ratio of 1:2 for HA-ALD:CS, or 1:1 for PEC-ALD:CS, with crosslinking occurring within seconds of mixing the components, yielding translucent gels.

### 3.7. Bionanocomposite Hydrogel Preparation

NsZnO powder was suspended in PBS and sonicated for 5 min at room temperature to disperse the NsZnO. The NsZnO suspension was added to the CS stock solution and gently stirred to ensure homogeneity. The previous formulations were tailored in order to get a final concentration of 1% wt of NsZnO, yielding opaque hydrogels.

### 3.8. Fourier Transform Infrared Spectroscopy

Infrared spectroscopy was carried out on a Thermo Scientific FTIR Spectrometer (Thermo Fisher Scientific, Morecambe, UK). Spectra were recorded for 16 scans in ATR mode at room temperature, with a 1 cm^−1^ resolution. Spectra were corrected for background and atmosphere using OMNIC (Windows 10 version) software (Thermo Scientific™) provided with the spectrometer. 

### 3.9. X-ray Diffraction

The pattern of the X-ray diffraction of the samples was obtained by using a PANalytical X’Pert Diffractometer (Cu Kα radiation, Almelo, The Netherlands). Data were collected with a 2D solid-state detector (PIXcel) from 20° to 60° (2θ) with a step size of 0.001° (2θ).

### 3.10. SEM-EDX

The freeze dried gels were analyzed by FESEM (Zeiss Supra 40, Carl Zeiss AG, Jena, Germany) equipped with an Oxford detector for energy dispersive X-ray analysis (EDX).

### 3.11. In Vitro Swelling Studies

The freeze-dried hydrogels were weighed and immersed in PBS solutions (pH 7.4) at 33 °C. At specific time points, the swollen hydrogels were removed and immediately weighed after the excess water on their surfaces was blotted away with filter paper, until the weight of hydrogels reached an equilibrium value. The swelling ratio (SR) was calculated as follows: SR (%) = ((*W_t_* − *W*_0_)/*W*_0_) × 100, where *W_t_* and *W*_0_ are the weights of the hydrogels in the swollen state and dry state, respectively. 

### 3.12. Cell Viability Studies

The cytotoxicity of the CS, HA-ALD and PEC-ALD was determined using a cell viability assay based on the metabolic activity of the cells as a measure of their viability and a proxy measure of cell proliferation. For this, 96-well plates were set up containing 100 μL of each compound at a starting concentration of 1 mg/mL. Cells were routinely maintained in Dulbecco’s modified Eagle’s medium (DMEM) with 10% fetal bovine serum and 1% antibiotics (10,000 μg/mL streptomycin and 10,000 units/mL penicillin) at 33 °C with 5% CO_2_. Cells were serially passaged or used for experiments at 70–80% confluence. Then, 10^4^ cells were added to each well, including control wells with medium alone. A set of control medium (100 μL of medium with serum) with no cells was also included. The 96-well plates were incubated at 72 h in a humidified incubator (5% CO_2_) at 33 °C. After incubation, the PrestoBlue™ assay was performed in accordance with the supplier’s guidelines. Briefly, 10 μL of PrestoBlue™ solution (Invitrogen, purchased from ThermoFisher, Morecambe, UK) was added to all wells and incubated for at least 30 min, after which the fluorescence was read using a Tecan Infinite^®^ 200 PRO platereader (fluorescence excitation/emission maxima: 560/590 nm) with Magellan software. After background (medium alone, no cells) subtraction, data were converted into the percent of untreated control (cells in medium). 

### 3.13. In Vitro Zn^2+^ Release

Zn^2+^ ion release from 200 µL of the bionanocomposite hydrogels was studied using vertical Franz diffusion cells and synthetic skin (Dow Corning 7-4107 Silicone Elastomer Membrane, supplied by Biesterfeld AG, Hamburg, Germany). HA-ALD/CS/NsZnO and PEC-ALD/CS/NsZnO were employed as donor phases. The receiving phase consisted of PBS. The apparatus was maintained at 33 °C with stirring; at scheduled times the receiving phase was withdrawn and entirely replaced with fresh receiving phase. Zinc ion quantification was performed for each sample using inductively coupled plasma mass spectrometry (ICP-MS, mod. 7500cc, Agilent Technologies, Milan, Italy) [63].

## 4. Conclusions

Here we report the facile synthesis of in situ crosslinking bionanocomposite hydrogels which deliver antimicrobial Zn^2+^ and have potential for wound healing applications [64,65,66,67,68,69,70]. The simple addition of extra components enables the inclusion of multiple drugs to treat wounds with various microbial populations (or indeed for patients with multiple co-morbidities). Such chemistry is appealing as the imines are inherently reversible and therefore impart the potential for self-healing [55] if the gel is exposed to mechanical forces (e.g., shear) before, during or after the initial gel formation reaction is complete, offering potential to include cells for cell therapy. The in situ crosslinking hydrogels presented here are attractive for application as injectable hydrogel-based tissue scaffolds that can adapt to fill patient-specific cavities, wherein the NsZnO particles are loaded with a variety of active pharmaceutical ingredients, and may therefore be of use for a variety of wound paradigms including those induced by extreme trauma (e.g., blast induced) [71,72,73].
