# Correction: Leone et al. In Situ Crosslinking Bionanocomposite Hydrogels with Potential for Wound Healing Applications. *J. Funct. Biomater.* 2019, *10*, 50

**DOI:** 10.3390/jfb16060212

**Published:** 2025-06-05

**Authors:** Federica Leone, Melike Firlak, Kirsty Challen, Wayne Bonnefin, Barbara Onida, Karen L. Wright, John G. Hardy

**Affiliations:** 1 Department of Chemistry, Lancaster University, Lancaster LA1 4YB, UK; federica.leone@polito.it (F.L.); m.firlak@gmail.com (M.F.); 2 Department of Biomedical and Life Sciences, Lancaster University, Lancaster LA1 4YG, UK; 3 Politecnico di Torino, Department of Applied Science and Technology, Corso Duca Degli Abruzzi 24, 10129 Turin, Italy; 4 Lancashire Teaching Hospitals NHS Trust, Emergency Department, Royal Preston Hospital, Sharoe Green Lane, Preston PR2 9HT, UK; kirsty.challen@lthtr.nhs.uk; 5 Advanced Medical Solutions Group PLC., Premier Park, 33 Road One, Winsford Industrial Estate, Winsford, Cheshire CW7 3RT, UK; wayne.bonnefin@admedsol.com; 6 Materials Science Institute, Lancaster University, Lancaster LA1 4YB, UK

## Error in Figure and Figure Legend

In the original publication [1], there was a mistake in Figure 3 and its legend. The X-ray diffraction data were collected using apparatus in Italy; we have replotted this in a single XRD figure using different software. The correct Figure 3 and legend appear below.

## Text Correction

There was an error in the original publication. The content has been changed since the X-ray diffraction data were collected using apparatus in Italy; we have replotted this in a single XRD figure using different software in Section 2 and Section 3.9.

- A correction has been made to Section 2, Paragraph 4:
  The X-ray diffraction (XRD) patterns (Figure 3) of HA-ALD and PEC-ALD are amorphous, and the gels formed from these components in the absence of NsZnO are correspondingly amorphous [56]. The XRD patterns of the bionanocomposite hydrogels containing NsZnO have peaks corresponding to NsZnO that are in agreement with our previously reported data [57], with clear evidence of a crystalline hexagonal phase with a wurtzite structure: the five main reflection peaks (100), (002), (101), (102) and (110) are consistent with those of the standard card for the hexagonal phase ZnO (JCPDS ICDD 36e1451). The inclusion of the NsZnO nanoparticles in the gels is clearly evident from the presence of the wurtzitic NsZnO XRD patterns in the bionanocomposite hydrogels. The preservation of the wurtzitic ZnO crystalline structure after the dispersion of the nanoparticles in the gel matrix is significant because of the link between structure and function [41]. Importantly, scanning electron microscopy (SEM) and energy-dispersive X-ray spectroscopy (EDS) confirmed the homogeneous dispersion of the NsZnO particles inside the polymeric matrix (Figure A2). This is of interest for the antibacterial activity of these biomaterials, as the reproducibility of the diffusion of Zn^2+^ will be dependent upon the homogeneity of dispersion within the gel matrix.
- A correction has been made to Section 3.9:
  The pattern of the X-ray diffraction of the samples was obtained by using a PANalytical X’Pert Diffractometer (Cu Kα radiation, Almelo, The Netherlands). Data were collected with a 2D solid-state detector (PIXcel) from 20° to 60° (2θ) with a step size of 0.001° (2θ).

The authors state that the scientific conclusions are unaffected. This correction was approved by the Academic Editor. The original publication has also been updated.

## Figures and Tables

**Figure 3:**
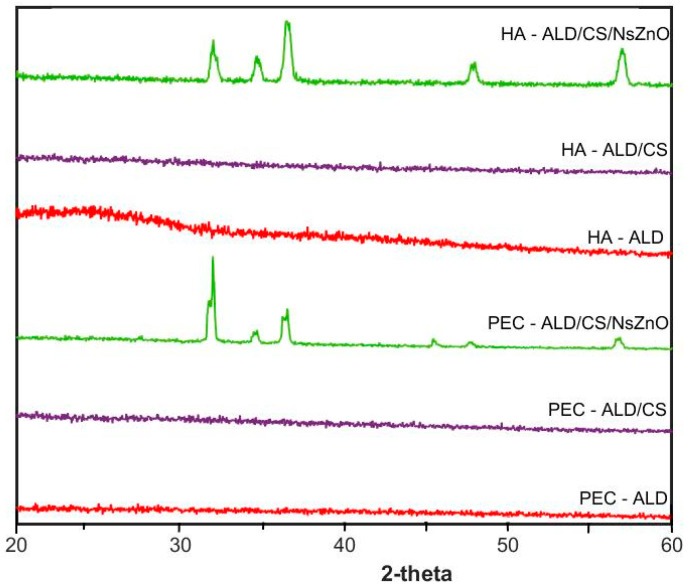
XRD patterns.

## References

[1] Leone F., Firlak M., Challen K., Bonnefin W., Onida B., Wright K.L., Hardy J.G. (2019). In Situ Crosslinking Bionanocomposite Hydrogels with Potential for Wound Healing Applications. J. Funct. Biomater..

